# White-Coat Hypertension: Pathophysiological and Clinical Aspects: Excellence Award for Hypertension Research 2020

**DOI:** 10.1161/HYPERTENSIONAHA.121.16489

**Published:** 2021-11-11

**Authors:** Giuseppe Mancia, Rita Facchetti, Michele Bombelli, Cesare Cuspidi, Guido Grassi

**Affiliations:** University of Milano-Bicocca (G.M.), University of Milano-Bicocca, Milan, Italy.; Clinica Medica, Department of Medicine and Surgery (R.F., M.B., C.C., G.G.), University of Milano-Bicocca, Milan, Italy.

**Keywords:** antihypertensive treatment, blood pressure, cardiovascular risk factors, goals, medicine, prognosis, therapeutics

## Abstract

Few issues of modern cardiovascular medicine have been as controversial as the relationship between white-coat hypertension (WCH), that is, a common condition in which office blood pressure is elevated while out-of-office blood pressure (ambulatory blood pressure or home blood pressure) is normal. While earlier studies showed no increased risk of cardiovascular events in WCH compared with the normotensive state, more recent studies have changed this conclusion by showing that an increased cardiovascular risk represents a trait of this hypertensive phenotype. The present article will review a number of issues related to WCH, that is, its definition, pathophysiological background, clinical alterations, and prognostic significance. This will be done by considering the available evidence published during the last decades, with special focus on the data collected in PAMELA (Pressioni Arteriose Monitorate e Loro Associazioni)—a research project performed with a cross-sectional and longitudinal design, which has provided a series of novel clinical information on WCH throughout the years. The final part of the article will discuss the therapeutic implications of the abovementioned evidence, as well as some controversial or still undefined issues related to WCH, whose investigation will be an important goal to pursue by future research.

White-coat hypertension (WCH) is known as a condition in which office blood pressure (BP) is elevated while out-of-office BP (ambulatory or home BP) is normal.^1–3^ After its identification almost 40 years ago, WCH has been the object of a large number of studies but also of different views about its clinical significance. For several years, the prevailing opinion has been that, compared with normotension, WCH carried no greater risk of cardiovascular outcomes and that thus its identification did not call for any further diagnostic or treatment measure.^4^ In the last 2 decades, however, this position has been weakened by studies that have almost invariably shown that WCH is associated with an unfavorable metabolic risk factor profile, a more frequent asymptomatic organ damage, and a greater risk of future progression to high cardiovascular risk conditions and cardiovascular morbid and fatal events. This has led to the conclusion that WCH is not clinically innocent—a position now shared by major hypertension guidelines.^1–3^

This article will review the clinical data that document, through various approaches, that WCH is prognostically less favorable than normotension. Although other key contributions will be duly mentioned, emphasis will be given to the results obtained in the Italian population sample of the PAMELA (Pressioni Arteriose Monitorate e Loro Associazioni) cross-sectional and prospective study because, although this study was considerably smaller than that based on data pooling from different cohorts,^5^ its design and measured variables allowed several important aspects of WCH to be suitably addressed. One, office, ambulatory, and home BP values were obtained in each subject within a restricted time, and all measurements were accurately standardized and of high quality, the number of the ambulatory BP measurements being uniformly high in all individuals throughout the daytime and nighttime. Two, the upper limit of ambulatory 24-hour (h) BP normality (specifically calculated from the PAMELA database) was lower (125/79 mmHg) than that used by other studies, that is, 130/80 mm Hg, respectively, or, in early investigations, even higher values. These more stringent thresholds for out-of-office BP normality reduced the chance of including in the WCH group truly hypertensive individuals, thereby guaranteeing a highly specific WCH identification. Three, data collection extended to metabolic risk factors and an echocardiographic evaluation was performed in each individual. And four, unlike other studies, the data collected initially were collected again 10 years later, and lethal events were registered over a long follow-up.^6–8^ Originally limited to untreated subjects,^9^ the definition of WCH has more recently been extended to patients under antihypertensive treatment, that is, those in whom treatment achieves BP control of out-of-office but not of office BP, known as white-coat uncontrolled hypertension or WUCH.^1^ Data on elevation of office but not of out-of-office BP in patients under antihypertensive drug treatment will also be discussed in the present article.

## Prevalence of WCH

WCH is a common condition. In the PAMELA study, individuals with an elevated office and a normal out-of-office BP were found to be about 15% of the general population and 30% to 40% of the hypertensive one, with no substantial difference when diagnosis was made via an office BP elevation vis-a-vis 24-h or home BP normality.^7^ The above figures are basically those reported for untreated subjects by recent hypertension guidelines,^1^ which also emphasize that, because the increase of systolic BP with age is steeper for office than for out-of-office BP,^10^ WCH is more common in the old fraction of the population, in which it may account for up to ≥50% of the hypertensive patients.^11^ The high prevalence of WCH emphasizes the importance for cardiovascular prevention of the research aimed at clarifying its pathophysiological aspects, clinical implications, and therapeutic needs.

## Dysmetabolic Risk Factors

**Figure 1.**
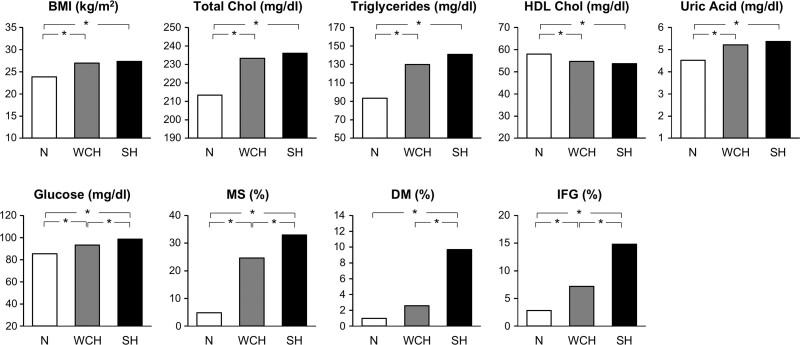
**Metabolic variables in normotensive (N), white-coat hypertensive (WCH), and sustained hypertensive (SH) subjects of the PAMELA (Pressioni Arteriose Monitorate e Loro Associazioni) population, that is, a sample of about 2100 subjects representative of the citizenship of Monza—a town in the northeast outskirt of Milan—for age decades (25–74 years), sex, and socioeconomic status.** Participation rate was 64% of the sample of 3200 subjects. The N status was established by office, 24-h, and home BP normality. WCH was diagnosed by office BP elevation (≥140 mm Hg systolic or 90 mm Hg diastolic) with a normal 24-h or home BP (<125/79 mm Hg or <132/82 mm Hg, respectively). SH was diagnosed by elevation of all 3 BP values. *P<0.05, statistical significance between groups. BMI indicates body mass index; Chol, cholesterol; DM, diabetes mellitus; HDL, high-density lipoprotein; IFG, impaired fasting glucose; and MS, metabolic syndrome. Data derived from Mancia et al.^8^

Although already reported by earlier investigations,^12^ the most complete description of the metabolic profile of WCH individuals is provided by the PAMELA study.^8^ As shown in Figure 1, compared with the normotensive group, WCH subjects (office BP ≥140 mm Hg systolic or 90 mm Hg diastolic, with a 24-h BP <125/79 mm Hg or a home BP <132/82 mm Hg) exhibited blood glucose, serum cholesterol, and serum triglyceride values lower than those exhibited by subjects with sustained hypertension (office and out-of-office BP elevation) but higher than those of normotensive subjects, that is, with office and out-of-office BP normality. This was the case also for the prevalence of conditions such as an increase of body mass index, an impaired fasting glucose, type 2 diabetes, metabolic syndrome and hypercholesterolemia, while HDL (high-density lipoprotein) serum cholesterol was progressively lower from normotension to WCH and sustained hypertension. In a more recent analysis of the PAMELA database, serum uric acid has also been found to significantly increase from normotension to WCH and sustained hypertension, strengthening the conclusion that in WCH glucose, lipid and other metabolic variables associated with cardiovascular risk^13^ are somewhat deranged from the values seen in individuals with a normal office and out-of-office BP pattern.

## Subclinical Organ Damage

Evidence has been repeatedly obtained that subclinical or asymptomatic alterations of organ structure and function are less common in normotension than in WCH, although remaining less frequent in the latter condition than in sustained hypertension. This is supported by the evidence collected in the PAMELA study that the prevalence of left ventricular hypertrophy (detected by a body surface area–calculated left ventricular mass index >99 and >114 g/m^2^, respectively, in women and men) was minimal in normotension (4.2%), intermediate in WCH (20.4%), and maximal in sustained hypertension (32.3%), with an adjusted (age and sex) odds ratio of exhibiting this condition that in WCH was 3.6 (95% CI, 2.2–5.9) and in sustained hypertension 4.3 (95% CI, 2.5–7.4) compared with subjects with office and out-of-office BP normality.^7^ Compared with normotension, WCH has also been shown to have a more frequent association with left ventricular diastolic dysfunction (reduced E/A ratio, where E refers to early ventricular relaxation and A to ventricular filling due to atrial contraction), left atrium enlargement, carotid intima-media thickening and plaques, increased urinary protein excretion, and silent cerebral infarction,^14–18^ which indicates that the WCH-related increase of organ damage is not limited to the heart but extends to other organs. This reflects an adverse effect of WCH on organ integrity over the preceding lifetime of the affected individuals. It also anticipates an adverse influence on the risk to experience future cardiovascular events because subclinical organ damage has been closely associated with an increase in the risk of overt cardiovascular outcomes,^9,19^ markedly raising the overall risk above the level calculated by the classical risk factor–based methods with no inclusion of silent organ damage measures.^20^

## Progression to High Cardiovascular Risk Conditions

**Figure 2.**
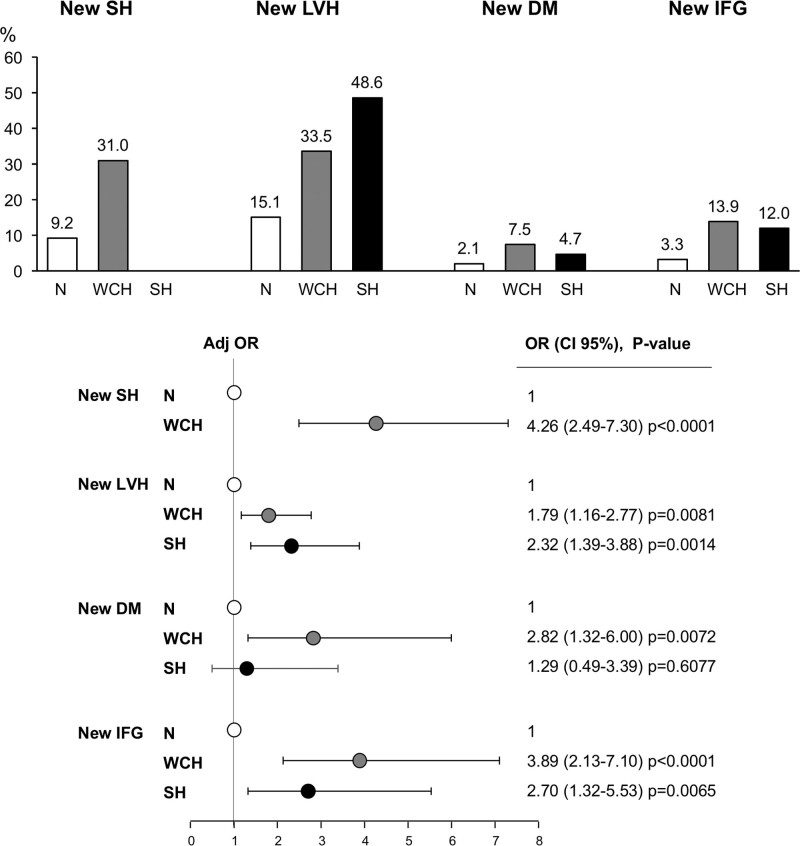
**Increase in incidence (top) and adjusted risk (bottom) of new-onset sustained hypertension (SH), new-onset echocardiographic left ventricular hypertrophy (LVH), new-onset diabetes mellitus (DM), and a new-onset impaired fasting glucose (IFG) state in the PAMELA (Pressioni Arteriose Monitorate e Loro Associazioni) population sample 10 y after the initial survey.** Data from normotensive (N), white-coat hypertensive (WCH), and limited to new LVH, DM, and IFG, from SH subjects. New LVH was identified by a left ventricular mass index >99 g/m^2^ in women and >114 g/m^2^ in men according to the body surface area; IFG was identified by a blood glucose ≥100 mg/dL, DM by a blood glucose ≥126 mg/dL or antidiabetic drug treatment and SH by addition of out-of-office to office BP elevation. Risk and *P* refer to age- and sex-adjusted data. Adj OR indicates adjusted odds ratio; and OR, odds ratio. Data derived from Mancia et al.^21–23^

A unique advantage of the PAMELA study is that subjects participating in the initial survey were seen again about 10 years later, allowing the same variables to be collected also after a long time interval. As shown in Figure 2 (top), compared with the initially normotensive group, WCH subjects exhibited, after 10 years, a greater incidence of new-onset sustained hypertension, that is, an elevation above normality also of out-of-office BP.^21^ They also exhibited a greater incidence of a new-onset impaired fasting glucose state, overt diabetes, and (in subjects with an initially normal left ventricular mass index) echocardiographic left ventricular hypertrophy.^22,23^ For all these new conditions, the adjusted risk associated with WCH was significantly greater than that of normotension, in some instances being similar to that exhibited by sustained hypertension. Thus, WCH is associated with a greater progression to a variety of high cardiovascular risk conditions, which makes the risk elevation with time greater than that due to aging alone. It is of additional interest that, over the 10 years between the first and the second survey, 24-h pulse pressure increased more markedly in WCH than in normotensive individuals (9.4±15.8 versus 5.9±13.2 mm Hg; *P*<0.05).^21^ Because a pulse pressure increase reflects a reduction of large artery distensibility,^1^ this suggests that over time, WCH individuals may exhibit a greater increase of large artery stiffening than that of subjects with normal office and out-of-office BP values.

## Cardiovascular Outcomes

**Figure 3.**
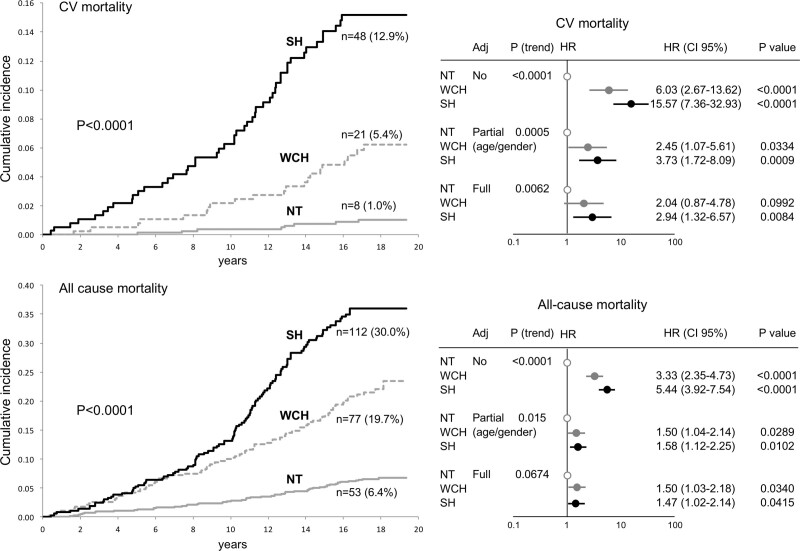
**Cumulative incidence (Kaplan-Meier curves) of cardiovascular (CV; upper left) and total mortality (lower left) in normotensive (NT), white-coat hypertensive (WCH), and sustained hypertensive (SH) subjects of the PAMELA study (Pressioni Arteriose Monitorate e Loro Associazioni.)** In the left panel, numbers refer to absolute and percent fatal events. The right panels show the corresponding hazard ratio (HR), having NT as reference. HR (and 95% CI) data are shown unadjusted, adjusted for age and sex, and after further adjustment for metabolic variables, smoking, previous CV events, antihypertensive treatment, etc (full). n refers to the number and percentage showing an event during the follow-up. Data derived from Mancia et al.^33^

At variance from earlier negative reports,^18,24^ the studies and the meta-analyses on the relationship between WCH, cardiovascular morbid or fatal events, and all-cause mortality published in the last 15 years have almost invariably shown that, mimicking the results obtained in the organ damage studies, an untreated WCH is associated with an incidence and risk of outcomes that is less than the risk associated with sustained hypertension but greater than the one shown by normotensive subjects.^25–35^ This has been the case also when the outcome risk was adjusted for sex and age, the age adjustment being made necessary by the greater prevalence of WCH in the elderly fraction of the population. As exemplified in Figure 3,^33^ it has also been the case when in the PAMELA study the risk of cardiovascular and all-cause mortality was quantified over a follow-up of about 16 years, and data were further adjusted for other potentially contributing cardiovascular variables, including antihypertensive treatment. This conclusion is in line with that of recent meta-analyses on the fully adjusted risk of a large number of untreated subjects followed for many years after the WCH diagnosis.^30–32^ In a meta-analysis of Huang et al on >50 000 untreated subjects,^31^ for example, WCH was found to be associated with a risk of cardiovascular events and total mortality that was, after adjustment for both demographic and baseline clinical variables, respectively 38% and 20% greater than that of normotension. A similar or greater increase in the fully adjusted risk of cardiovascular events (36%) and total mortality (33%) was reported for WCH by another even larger meta-analysis,^32^ albeit with the limitation that the study majorly contributing to the final data was later retracted because data analysis had been found to be inaccurate.^36^ In this context, however, it is important to mention that quantification of the risk of WCH by adjustments that extend beyond age and sex and neutralize the role of dysmetabolic and other alterations associated with this condition is a somewhat controversial procedure^8,29,37^ because of its interference with the prognostic significance of WCH as a multifactorial clinical entity. In other words, such an extensive adjustment, although justified when the purpose is to try to determine the role of the BP values of WCH in the overall cardiovascular risk of this condition (see below), leads to an amputation of the other adverse phenotype traits of WCH and thus to an underestimation of its prognostic significance as a clinical entity. In clinical practice, this is the first important information to be provided to physicians who have to decide how closely to follow these patients over time and whether and to what intensity treatment of risk factors is necessary, that is, decisions dependent on a correct estimate of total cardiovascular risk.

## Cardiovascular Outcomes, Out-of-Office, and Office BP

**Figure 4.**
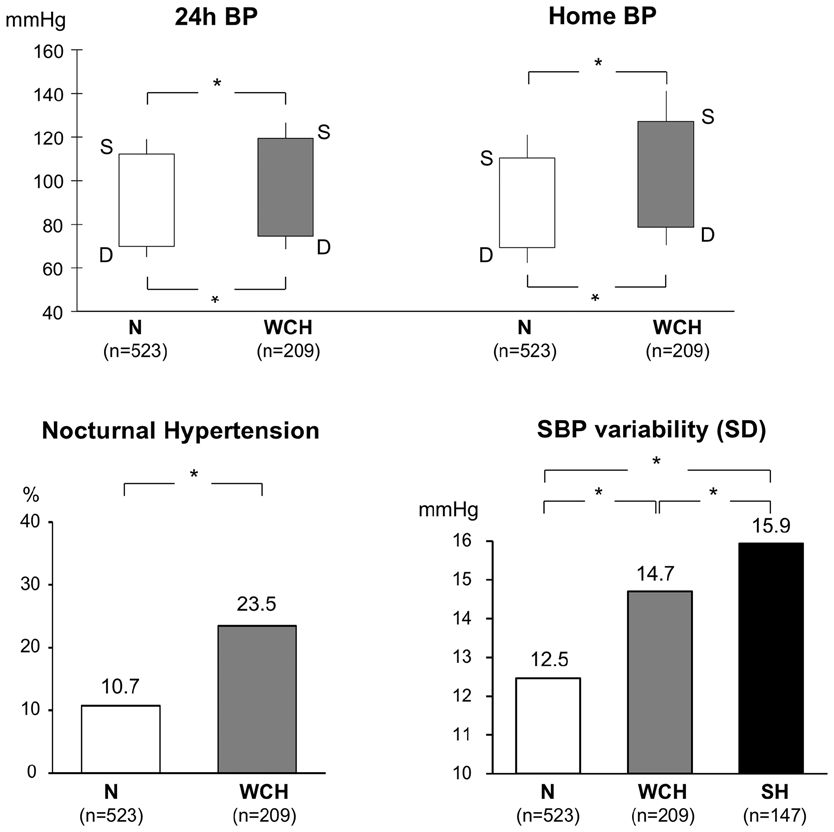
**Twenty four–hour and home systolic (S) and diastolic (D) blood pressure (BP) values in normotensive (N) and white coat hypertensive (WCH) subjects from the PAMELA (Pressioni Arteriose Monitorate e Loro Associazioni) population.** **Bottom**, **Left**, Prevalence (%) of nocturnal hypertension in N and WCH. **Bottom**, **Right**, SBP variability in N, WCH, and sustained hypertension (SH) of the PAMELA study. *P* refers to data adjusted for age and sex. Twenty-four-hour BP variability was calculated as the variability value that survived the elimination of the oscillatory variability components (day-night and prepostprandial) identified by the Fourier analysis, which was termed residual or erratic variability. Data derived from Mancia et al,^33^ Cuspidi et al,^39^ and Mancia et al.^8^

As mentioned above, full adjustment for demographic and clinical variables is not devoid of a useful role because persistence of a greater risk of cardiovascular outcomes and mortality after full adjustment for demographic and clinical variables (other than BP) implies that in WCH, the increase of morbid and fatal events is causally also dependent on the WCH BP pattern. In this context, several pieces of evidence support the involvement of out-of-office BP. First, although normal by definition, ambulatory and home BP are higher in WCH than in normotensive controls. As shown in the upper panel of Figure 4, in the PAMELA study, the systolic BP difference between the WCH and normotensive groups was 7.1 mm Hg (119.4 versus 112.3 mm Hg) for the 24-h BP values and 16.7 mm Hg (127.2 versus 110.5 mm Hg) for the home BP values,^33^ the corresponding differences in diastolic BP being 4.5 and 9.4 mm Hg. Substantial differences have also been reported in a large meta-analysis of available studies.^31^ This is clinically relevant because an increase of 24-h or home BP is associated with an increase of cardiovascular mortality not only when the background out-of-office BP is elevated but also when, as in WCH, out-of-office BP lays within the normal range. In the PAMELA population, for example, a 10-mm Hg increase of 24-h or home systolic BP above values around 120 to 130 mm Hg has an adverse impact on cardiovascular mortality.^38^ Furthermore, compared with normotension, WCH has been found to be more frequently accompanied by nocturnal hypertension (defined as nighttime mean BP values above those representing nocturnal BP normality^1,2^), which in this condition can have an age- and sex-adjusted prevalence of about one-third of the entire WCH group^39^ (Figure 4, bottom left). This is also clinically relevant because several studies have found that nocturnal BP carries a greater adverse prognostic significance than that of daytime BP.^40–43^ Finally, the PAMELA data show that in WCH, 24-h systolic BP variability is less than in sustained hypertensive subjects but greater than in normotensive controls (Figure 4, bottom right).^8^ Because 24-h BP variability increases cardiovascular risk independently on 24-h mean BP values,^44–47^ this adds another potential factor to those suggesting a role of out-of-office BP on the increased cardiovascular risk seen in WCH after extensive adjustment for clinical confounders.

It should be mentioned that the above described participation of out-of-office BP to the cardiovascular risk of WCH does not exclude a causal role of office BP as well. In the PAMELA cohort, we have seen that office BP was one of the predictors of the development of new-onset sustained hypertension, independently on other risk factors, including out-of-office BP.^48^ Furthermore, WCH showed a greater increase in the fully adjusted risk of cardiovascular mortality over the normotensive risk value if office BP showed an elevation above 140/90 mm Hg at 2 consecutive visits (one before and another after the only available 24-h ambulatory BP monitoring), compared with subjects in whom the office BP elevation was found in one of the 2 visits only.^49^ Thus, in WCH, cardiovascular risk may be adversely modulated by both the in- and out-of-office components of the BP pattern of this condition. One possible explanation is that office BP values reflect a hyperreactivity to stressful stimuli, which have been associated with the genesis of hypertension and cardiovascular complications in experimental^50,51^ and some human studies.^52,53^ However, in humans, the reactivity to different stressors can be so discrepant^54^ as to make difficult to enclose it in a single comprehensive measure of stressor-related BP reactivity. Furthermore, no association has been found between the directly measured increase of office BP during a doctor visit and 24-h BP variability,^55^ which has been regarded as a hypertensiogenic factor. Finally, the so-called white-coat effect as measured by the office-daytime BP difference has never convincingly been associated with the risk of cardiovascular events.^56,57^ The factors responsible for the office BP modulation of cardiovascular risk in WCH, therefore, remain to be clarified.

## Subgroup Heterogeneity of Cardiovascular Risk

Pooling data from several cohorts, the International Database of Ambulatory BP in Relation to Cardiovascular Outcomes (IDACO) has reported that WCH was associated with a cardiovascular risk increase if subjects were old, cardiovascular risk was high, or the office BP elevation was limited to systolic values.^58^ In contrast, in younger patients, cardiovascular risk was not found to be significantly different from that of normotensive controls.^58^ This implies that WCH may have a different prognostic significance in different groups according to their different demographic and clinical background. This is a reasonable, and to some extent obvious, possibility although, as far as age-related cardiovascular risk differences are concerned, a caveat may be that proving that WCH is associated with an increased risk in young or low-risk individuals is difficult because (1) the lower incidence of events limits the statistical power of group comparisons and (2) at a younger age, disease may progress for many years as an increase of silent organ damage rather than as the appearance of an overt clinical event. This is beyond the possibility to be addressed by the IDACO and most other studies in which measures of organ damage are not available. It should also be mentioned that in large meta-analyses, WCH has shown an association with an increased risk of cardiovascular events and mortality both in subjects aged <55 and in those aged ≥55 years.^31^ This is in line with the conclusion of the PAMELA study in which the average age of the cohort was 52.7 years and recruitment was based on the balanced representation of decades between 25 and 74 years, making the data not predominantly representative of an older age.^38^

## Risk Discrimination in WCH Individuals

Regardless the possible differences of cardiovascular risk in different WCH subgroups, the risk of WCH obviously varies at the individual level, which is why guidelines recommend accurate risk quantification in each WCH subject as a guide to decide on follow-up, lifestyle, and also BP and other risk factor–based treatment measures.^1–3^ This quantification can be obtained by careful collection of clinical history, thorough assessment of metabolic risk factors, and in-depth search for structural and functional organ alterations.^20^ The PAMELA study, however, has identified 3 additional diagnostic possibilities that can be implemented at a practical level. One possibility is to obtain BP data from either ambulatory and home BP measurements, given that in about 40% of the PAMELA population, 24-h BP normality was found to be accompanied by home BP elevation and vice versa.^33^ The incidence of all-cause mortality was 13.4% in individuals in whom the two out-of-office BP values were both normal versus 24.2% in those in whom only one out-of-office BP was normal while the other was elevated, the corresponding incidence for cardiovascular mortality being 1.2% and 13.4%. Compared with normotensive subjects, the age- and sex-adjusted risk (hazard ratio) of all-cause and cardiovascular mortality (1.31 and 0.77, respectively) was not significantly greater in WCH subjects with normal home and 24-h BP, whereas in WCH subjects in whom only one out-of-office BP was normal, both age- and sex-adjusted hazard ratio values showed a significant increase (all-cause mortality, 1.59 [95% CI, 1.08–2.36]; *P*=0.02; cardiovascular mortality, 3.26 [95% CI, 1.40–7.59]; *P*<0.0001). In these WCH subjects, hazard ratio for all-cause and cardiovascular mortality was significantly greater than in normotensive subjects also when data were adjusted not only for demographic but also for clinical variables other than BP that could potentially contribute to the mortality risk, such as serum lipids, blood glucose, history of cardiovascular events, antihypertensive treatment, etc.^48^ Another possibility is to obtain a second set of out-of-office and office BP measurements because replication at a second visit of an office BP elevation on one side or of a 24-h BP normality on the other has been found to be associated with a greater risk of cardiovascular events in the first case and a lower prevalence of cardiac, vascular, and renal damage in the second.^49,59^ A third possibility is to pay attention to the BP values because in the WCH subjects of the PAMELA study, the risk was found to be greater in WCH subjects in whom 24-h BP was greater than the median value of the WCH group as a whole, after adjustment for office BP. This was the case also for an above-the-median office BP, after adjustment for 24-h BP (unpublished observations). These data imply that WCH is not a yes or no condition but rather a condition in which cardiovascular risk is quantitatively modulated by both components of its BP pattern.

The above description provides guidance on what should be diagnostically done in patients in whom office and out-of-office BP measurements identify a WCH condition. One, patients’ history should be carefully collected and metabolic risk factors carefully measured. Two, subclinical damage should be searched for in different organs because cardiovascular risk has been shown to increase in parallel with the number of organs affected^20^ and even with different measures of the damage within the same organ, such as, in the kidney, microalbuminuria, and reduction of glomerular filtration rate.^60^ Search should make use of an EKG and an echocardiogram (to visualize alterations of cardiac structure, myocardial-strain, and systolic and diastolic dysfunction), urinary protein excretion and glomerular filtration rate estimation, and an echo Doppler–based examination of the carotid arteries to visualize intima-media thickening and plaques. Measurement of pulse wave velocity may be also useful although its changes in WCH have been less well studied and a stiffening may reflect an alteration of large artery anatomy but also a passive response to the BP increase. Three, measurements of office and out-of-office BP should be accurate and obtained more than a single time. Four, information should extend to both home and ambulatory BP values, rather than being limited to one type of out-of-office BP, as currently done in clinical practice. Ambulatory BP measurements, in particular, should be collected via a series of measurements that allow good quality information on nighttime BP values, given the prognostic importance of nocturnal BP and its frequent abnormality in WCH.

## Treatment

**Figure 5.**
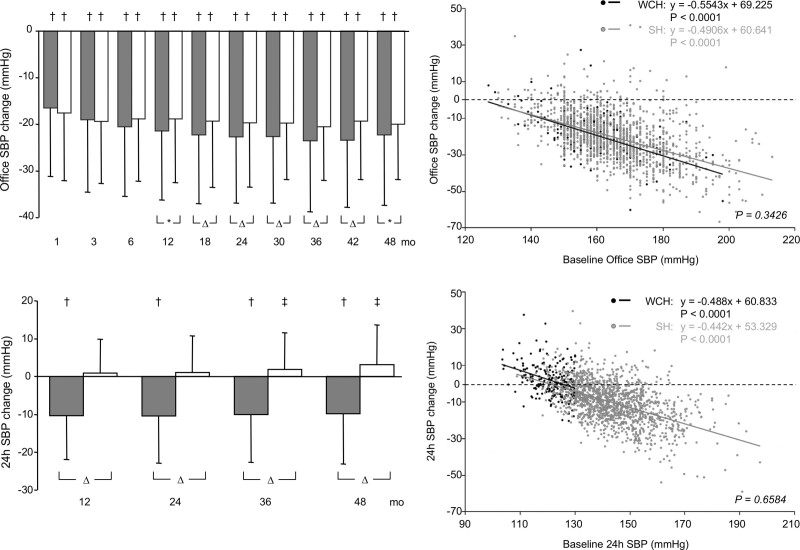
**Reduction of office and 24-h systolic blood pressure (SBP) in the hypertensive patients of the ELSA trial (European Lacidipine Study on Atherosclerosis) treated with antihypertensive drugs for 4 y.** SBP was measured at narrow intervals during the initial titration phase and then at intervals of 6 mo. Grey histograms refer to sustained hypertensive patients and white histograms to white-coat hypertensive (WCH). The right panels show the relationship between the treatment-induced reduction of office or 24-h SBP and the corresponding baseline values. White points indicate WCH patients and grey points, sustained hypertensive (SH) patients. Statistical significance between groups are shown by the following symbols: *P<0.05, Δ*P*<0.01, † and ‡*P*<0.0001 vs control. WCH indicates white-coat hypertension. Data derived from Mancia et al.^61^

There is general agreement that because of the greater prevalence of metabolic risk factors, as well as of the risk of future sustained hypertension, diabetes, and organ damage, WCH individuals should be offered a closer follow-up (and frequent out-of-office BP measurements) and submitted to correct lifestyle measures.^1–3^ In contrast, lack of suitable evidence prevents any evidence-based agreement on whether WCH deserves antihypertensive drug treatment, which is thus the Achilles’ heel of the information on this condition. The BP effects of antihypertensive drug treatment in WCH have been satisfactorily addressed in ELSA (European Lacidipine Study on Atherosclerosis) in which a calcium channel blocker or a β-blocker was given (with the addition of a diuretic, if needed) for 4 years to about 2200 hypertensive patients in whom office and ambulatory BP were measured at an interval of 6 and 12 months, respectively.^61^ As shown in Figure 5, treatment lowered BP almost as effectively and consistently in WCH and sustained hypertension. This was not the case for 24-h BP, however, which decreased effectively throughout the duration of the study in sustained hypertension while showing a small tendency to increase from the first to the last year of treatment in WCH (Figure 5, bottom left), an effect that was similar when data were analyzed separately for the two different treatment strategies. This can probably be explained by a phenomenon such as regression to the mean in subjects in whom baseline 24-h BP values were low. In contrast, an explanation based on the inability of antihypertensive drugs to lower ambulatory BP in WCH is made unlikely by the observation that the relationships between baseline and on-treatment office or 24-h BP were superimposable over the entire range of the 2 BP values in WCH and sustained hypertension (Figure 5, right).^61^ At any rate, at a practical level, antihypertensive drug administration to WCH subjects can be expected to effectively lower office while having much less effect on ambulatory BP, although some ambulatory BP reduction is likely to occur if baseline 24-h BP values are in the high normality range.^61^

**Figure 6.**
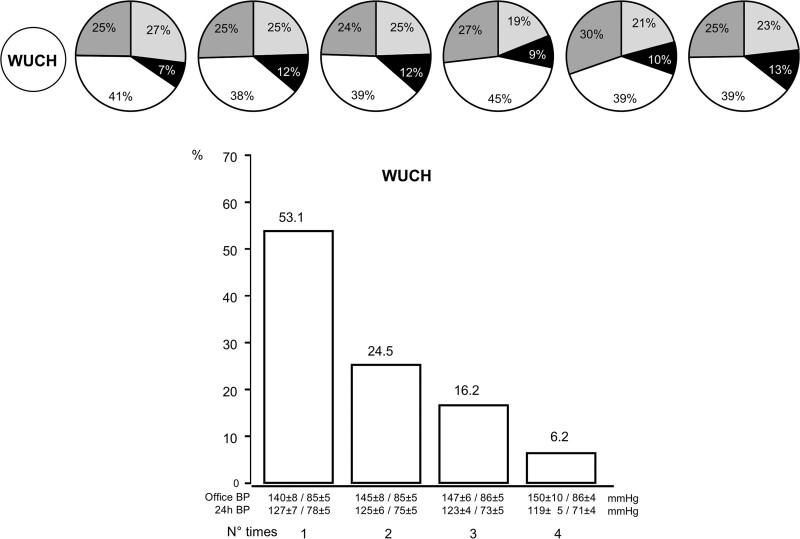
**Percentage of patients with white-coat uncontrolled hypertension (WUCH) at the first set of office and ambulatory blood pressure (BP) measurements who, ≥1 y later, maintained the same status (white color) or became uncontrolled hypertensives (no office and 24-h BP control, dark grey), masked hypertensives (no 24-h BP control, black) or controlled hypertensives (both office and 24-h BP control, light grey).** Histograms below the circles show how often WUCH was detected during the treatment period, that is, 1, 2, 3 or all 4 times BP measurements were made (yearly intervals). Data derived from Mancia et al.^63^

No randomized placebo-controlled trial has ever addressed whether BP-lowering drug treatment has an effect on the increased risk of outcomes of WCH individuals. On this background, however, an ambulatory BP substudy of the Syst-Eur trial (Systolic Hypertension in Europe) is regarded as indicating no treatment benefit because in WCH patients, the number of events was not significantly different in the treated and placebo groups.^62^ A negative significance is also ascribed to longitudinal studies and meta-analyses on patients in whom antihypertensive treatment achieved 24-h but not office BP control, the so-called WUCH patients, in whom the results do not show any greater cardiovascular or mortality risk compared with treated patients with an in- and out-of-office BP control.^31,32^ However, in the Syst-Eur substudy, only 6 and 2 events were available in the placebo and treatment groups, respectively, which makes its negative conclusion weak. Also, in studies on treated patients, WUCH identification was based on a single set of office and out-of-office BP values, usually in an early treatment phase. This is also a major limitation because, in the ELSA trial, analysis of the office and ambulatory BP measurements obtained every year during a 4-year antihypertensive treatment has shown that WUCH is an inconsistent condition, that is, that from 1 year to the next one, most treated patients move from WUCH to other conditions, such as control of in- and out-of-office BP, lack of control of in- and out-of-office BP, or even a phenotype that can be regarded as opposite to WUCH, that is, control of office but not of out-of-office BP by treatment or MUCH (Figure 6).^63^ In the analysis of the ELSA data, only 4.5% of the WUCH patients exhibited the same condition throughout the 4 years of the trial (Figure 6).^63^

The problem of WCH and antihypertensive treatment deserves a final consideration. Because of its prevalence, WCH was presumably common in trials that have documented the protective effect of antihypertensive treatment, as well as its relationship with the magnitude of the office BP reduction.^64,65^ This might have been especially the case in mild-to-moderate hypertension and in hypertension of the elderly in which WCH accounts for about 30% to 50%, of their overall prevalence.^1,11^ It seems unlikely that the documented protective effect of treatment in these conditions could have been reached without involvement of the WCH fraction of the trial population. This legitimates the opinion that, until evidence for the contrary is obtained, WCH should not be denied BP-lowering interventions.^8^

## Is WCH an Appropriate Terminology?

The term WCH implies that the increase of office but not of out-of-office BP originates from a pronounced alerting-dependent BP increase, which is known to occur in the doctor’s office^55^ but not when BP is measured automatically or semiautomatically in or close to real-life conditions.^1^ However, a look into the available data does not entirely support this mechanistic explanation. First, an alerting response to the doctor’s measurement of BP includes an increase of heart rate,^55,66,67^ which is hardly compatible with the similar or only marginally greater heart rate values that characterize WCH compared with normotensive subjects. Second, the larger prevalence of WCH in elderly individuals is not accompanied by a greater response of the elderly to stress or a doctor’s visit, as it should be if a greater alerting response was the responsible factor. Indeed, cardiovascular responses to stress do not appear to increase with age,^54^ and the difference between office and out-of-office BP has not been found to quantitatively reflect or correlate with the WC effect,^56^ as directly measured during a doctor’s visit in subjects under intra-arterial or noninvasive beat-to-beat BP measurements. This does not exclude participation of an emotional factor in the genesis of WCH, but it suggests that other factors are probably also involved. Search for these factors has probably been slowed down by the popularity reached by the explanation based on the role of an alerting response, against which other more etiologically neutral and descriptive terminologies have been unsuccessful. A thorough characterization of the WCH genotype and phenotype, in connection with and independently from their alerting component, is desirable, however, to better identify the reasons for the abnormalities associated with this condition. Evidence has been obtained that, compared with normotension, WCH is accompanied by an increased peripheral sympathetic nerve traffic that is not significantly different from that exhibited by sustained hypertension.^68–70^ This has prognostic implications because a sympathetic overdrive enhances organ damage and increases mortality in a number of diseases.^71^

## Unmet Needs and Conclusions

Although there is now a general agreement that WCH has a greater cardiovascular risk than normotension, evidence is absent, limited, or controversial on several important aspects of this condition, which makes further appropriate studies necessary. A most important study would be to perform a randomized, placebo-controlled trial on the effect of BP-lowering treatment on cardiovascular outcomes in WCH, to see whether a BP reduction protects subjects affected by this condition and provide guidelines with material for evidence-based treatment recommendations in a large fraction of the hypertensive population. Such a trial will have to consider that in WCH, the overall incidence of cardiovascular outcomes is limited, which means that a large number of patients and a long follow-up will be necessary. This problem will be attenuated by the inclusion, among the end points of the treatment-induced improvement, of organ damage for which there is evidence of an association with cardiovascular outcome reduction, such as regression of left ventricular hypertrophy and urinary protein excretion.^72,73^ It will also be important to improve the quality of the observational studies on WCH, which are almost always based on its identification by a single set of office and out-of-office BP measurements, the results being then used to see the WCH ability to predict events over many years. Unfortunately, the poor reproducibility of WCH (see above) makes single detection of WCH a serious limitation. Studies based on multiple office and out-of-office BP measurements are necessary to avoid looking at the long-term prognostic significance of what might be just an occasional BP pattern. This is particularly necessary in patients under antihypertensive treatment because in this condition, the chance for a single detection to reflect the WCH persistency over many years of follow-up is made even lower by the frequency of the treatment changes and the low and variable adherence to treatment prescriptions. Other important studies on WCH would be (1) to compare the association of WCH and masked hypertension with cardiovascular outcomes, the latter being regarded as having a greater risk, and thus to need treatment, based on uncertain evidence, (2) to better define the factors involved in the adverse prognostic role of office BP in WCH, and finally, (3) to clarify, by long-term observational studies or organ damage-based trials, the clinical significance of WCH in younger patients, at early stages of hypertension or when asymptomatic organ damage is still absent.

## Article Information

### Sources of Funding

None.

### Disclosures

None.
